# Current view of liver cancer cell-of-origin and proposed mechanisms precluding its proper determination

**DOI:** 10.1186/s12935-022-02843-0

**Published:** 2023-01-06

**Authors:** Tomasz Gromowski, Veronika Lukacs-Kornek, Jaroslaw Cisowski

**Affiliations:** 1 Department of General Biochemistry, Faculty of Biochemistry, Biophysics and Biotechnology, Jagiellonian University, Krakow, Poland; 2 Institute of Experimental Immunology, University Hospital of the Rheinische Friedrich-Wilhelms-University, Bonn, Germany

**Keywords:** Hepatocellular carcinoma, Cholangiocellular carcinoma, Differentiation, Cell plasticity, Cell-of-origin, Cancer stem cells, Transdifferentiation, Metaplasia, Epithelial-to-mesenchymal transition

## Abstract

Hepatocellular carcinoma and intrahepatic cholangiocarcinoma are devastating primary liver cancers with increasing prevalence in many parts of the world. Despite intense investigation, many aspects of their biology are still largely obscure. For example, numerous studies have tackled the question of the cell-of-origin of primary liver cancers using different experimental approaches; they have not, however, provided a clear and undisputed answer. Here, we will review the evidence from animal models supporting the role of all major types of liver epithelial cells: hepatocytes, cholangiocytes, and their common progenitor as liver cancer cell-of-origin. Moreover, we will also propose mechanisms that promote liver cancer cell plasticity (dedifferentiation, transdifferentiation, and epithelial-to-mesenchymal transition) which may contribute to misinterpretation of the results and which make the issue of liver cancer cell-of-origin particularly complex.

## Background

Hepatocellular carcinoma (HCC) and intrahepatic cholangiocarcinoma (iCCA) are two types of primary liver cancer (PLC) with increasing incidence worldwide and high death rate. HCC is responsible for most PLC cases, while iCCA accounts for approximately 10% of the cases. The incidence of PLC is highest in developing countries; primarily in Southeast Asia and sub-Saharan Africa. In Europe, the southern part has higher incidence than the northern part [1–3]. The grim outcome for patients with PLC stems from the usual late diagnosis, the lack of recurrent, targetable molecular alterations, and poorly understood biology, which precludes the development of effective therapies.

HCC and iCCA exhibit different histopathological features, closely similar to hepatocytes and cholangiocytes, respectively. For this reason, they have long been considered exclusively derived from these distinct cell lineages. Interestingly, however, HCC and iCCA share some risk factors, most prominently chronic hepatitis B virus (HBV) and hepatitis C virus (HCV) infections, alcohol consumption, cirrhosis, obesity, diabetes, nonalcoholic fatty liver disease (NAFLD), and possibly tobacco smoking [3–8]. Moreover, some common molecular alterations, e.g. mutations and deletions in potentially driving oncogenes involved in chromatin modification (inactivating mutations in *Arid1a*), protein deubiquitination (*Bap1*), cell cycle regulation (*Cdkn2a*, *Cyclin D1*, *Cyclin A*, *KRAS*), PI3K signaling (*PIK3CA*, *PTEN*) or secretory proteins (*Albumin*), are frequently found in both HCC and iCCA [9–11]. Accordingly, in combined HCC-iCCA, a subtype of PLC with a very poor prognosis, both components usually contain the same mutational signatures suggesting the common cell of origin [12–14].

**Fig. 1:**
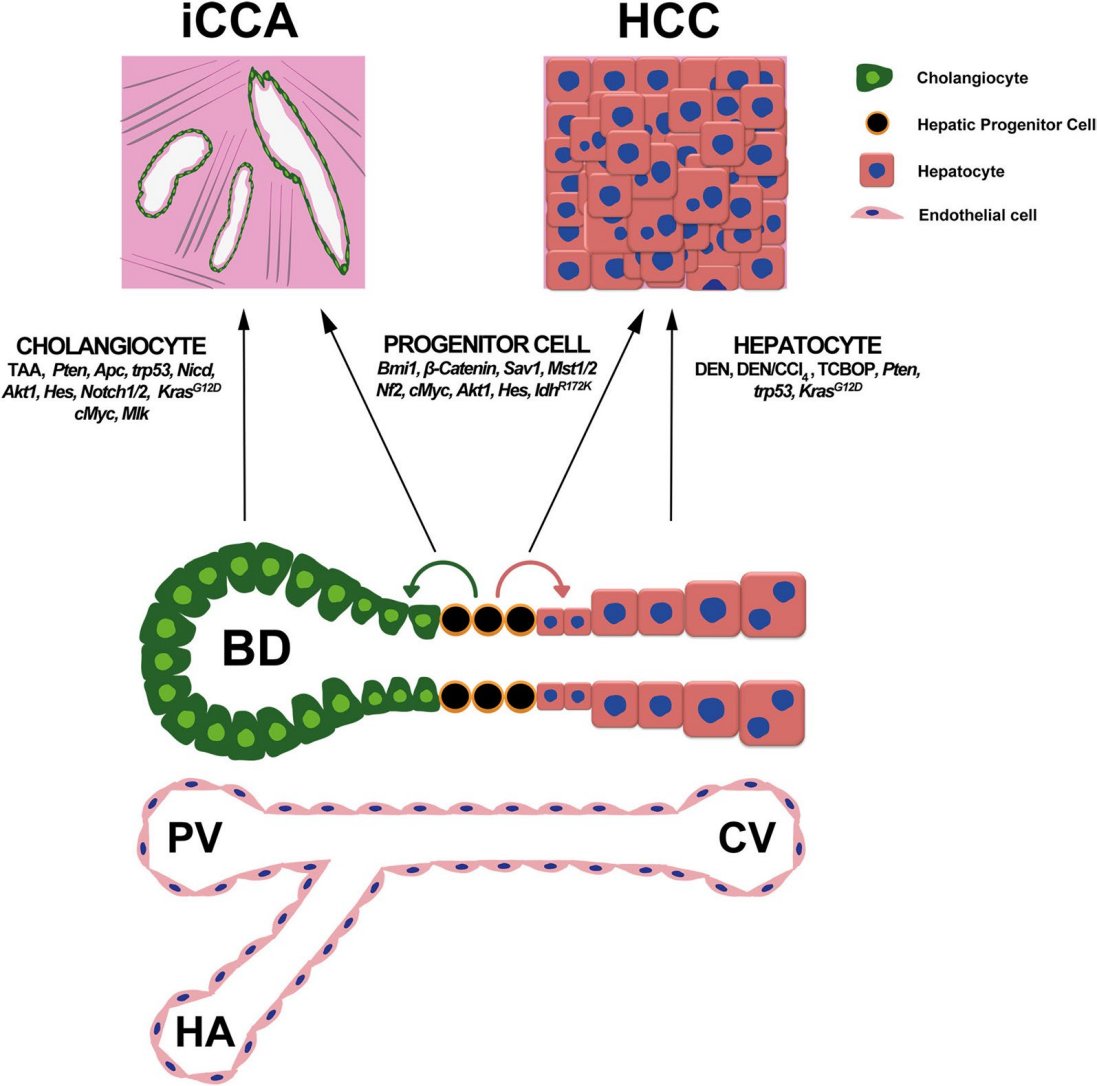
Summary of the evidence supporting the contribution of different cell lineages as CoO in HCC and iCCA. In a healthy liver, hepatic progenitor cells give rise to both cholangiocytes and hepatocytes. Cholangiocytes form bile ducts close to the portal vein (PV) and hepatic artery (HA), whereas hepatocytes constitute the bulk of liver mass and during their life cycle migrate towards the central vein (CV). Data from rodent models collected over the years suggest that any type of epithelial cell present in the liver (hepatocytes, cholangiocytes, and hepatic progenitor cells) can serve as CoO depending on the initiating event. Hepatocytes treated with chemical carcinogens, DEN, DEN/CCl_4_ or TCPOBOP acquire mutations and undergo malignant transformation toward HCC. Similarly, transformation of cholangiocytes with TAA results in generation of iCCA. Hepatic progenitor cells may undergo malignant transformation following genetic events. *BD* bile duct; *CCl*_*4*_ carbon tetrachloride, *CV* central vein, *DEN* Diethylnitrosamine, *HA* hepatic artery, *PA* portal vein, *TAA* thioacetamide, *TCPOBOP* 3,30,5,50-tetrachloro-1,4-bis(pyridyloxy)benzene

Altogether, these observations suggest that HCC and iCCA may, at least under some circumstances, be derived from the same cell-of-origin (CoO). In principle, the purported CoO could be an oncogenically transformed hepatocyte, cholangiocyte, or their common progenitor (bona fide cancer stem cell); the contribution of other liver-resident cell types (e.g., Stellate cells, Kupffer cells, endothelial cells, and fibroblasts), although cannot be excluded in principle, has not been demonstrated thus far. Furthermore, one report published recently has attributed bone marrow-derived cells which immigrated to the liver with HCC initiating properties [15], the finding which requires confirmation in the future. However, the formal and experimental determination of CoO in liver cancer has been precluded by the paucity of techniques enabling the clear identification of cell lineages that are the primary targets of oncogenic hits, not just their progenies. This issue has been additionally complicated by dedifferentiation, transdifferentiation, and epithelial-to-mesenchymal transition (EMT), processes which have been shown to enable acquisition (at least transitory) of traits characteristic of different cell lineages. The dissection of the CoO in PLC has only become possible with the advent of the genetic lineage tracing approach in mice. The purpose of this review is to summarize and evaluate the evidence supporting the role of hepatocytes, cholangiocytes and progenitor cells as CoO in liver cancer and to highlight possible mechanisms responsible for changing cell identity, a process that may confound prudent determination of CoO in liver cancer (summarized in Fig. 1 and Table 1).

**Table 1:** Summary of data supporting the contribution of different cell types to the origin of liver cancer

Liver tumor type	Purported CoO	Mouse model	Approach	References
HCC	Hepatocyte	*Sox9-Cre*^*ERT*^; R26R^*YFP*^; *MUPuPA/*HFD; STZ/HFD	DEN; GEMM; lineage tracing	[35]
		*Opn-Cre*^*ERT2*^; *K19-Cre*^*ERT2*^; *R26R*^*YFP*^; AAV8-Tbg-Cre; *Rosa26*^*loxP-mTom-stop-loxP–mGFP*^ *Rosa26*^*loxP-stop-loxP–ZsGreen1*^ *Mdr2*^KO^; *Pten*^*flx/flx*^	DEN/CCl_2_; CDE or DDC diet; GEMM; lineage tracing	[38]
		*hURI-tetOFF*^*hep*^; *(SA)Cre*^*ERT2*^ *R26R*^*YFP*^; *AAV8-Tbg-Cre*; *Rosa26*^*loxP-mTom-stop-loxP–mGFP*^ *Sox9-Cre*^*ERT*^; *K19-Cre*^*ERT2*^* R26R*^*ZsGreen*^	DEN/CCl_2_; GEMM; lineage tracing	[41]
		*Hnf1b-Cre*^*ER*^; R26R^*Tom*^; *Mdr2*^KO^	DEN; GEMM; lineage tracing	[44]
		*AAV8-Tbg-Cre*; *Foxl1-Cre*; *R26R*^*YFP*^	DEN/CCl_2_; TCPOBOP GEMM; lineage tracing	[45]
HCC; iCCA		CMV-SB; *TREt-Yap-IRES-GFP*; *EF1a-miR-30*; *EF1a-rtTA3-shp53*; *Cre-ER*; *trp53*^*flx/flx*^; *EF1a-rtTA3-shAPC*; *EF1a-NICD-IRES-mCherry*	DEN; GEMM; HTVI; lineage tracing	[51]
iCCA		CMV-SB; *EF1a-HAmyrAKT1*; *EF1a-MycNICD*; *AAV8-Tbg-Cre*; *R26R*^*YFP*^	GEMM; HTVI; lineage tracing	[53]
		*Alb-Cre*^*ERT2*^; *K19-Cre*^*ERT2*^; *R26R*^*Notch*^; R26R^*YFP*^; R26R^*YFP*^; *Hes *^*flx/flx*^	TAA; GEMM; lineage tracing	[60]
HCC; iCCA		*CMV-SB*; pT3-*EF1a-HAmyrAKT*; pT3-*EF1a-Yap*^*S127A*^; pT3-EF1α-dnRBPJ; AAV8-Tbg-Cre; R26R^*YFP*^; *Notch1 *^*flx/flx*^; *Notch2 *^*flx/flx*^;	HTVI; GEMM; lineage tracing	[61]
iCCA	Cholangiocyte	*CK19-Cre*^*ERT2*^; *R26R*^*YFP*^; *trp53*^*flx/flx*^	TAA; GEMM; lineage tracing	[67]
HCC; iCCA	Cholangiocyte or hepatocyte	*Alb-Cre*^*ERT2*^; *Alb-Cre*; *CK19-Cre*^*ERT2*^; *Pten*^*flx/*+^; *Pten*^*flx/flx*^; *LSL-Kras*^*G12D*^; *Rosa26*^*loxP-mTom-stop-loxP–mGFP*^	GEMM; lineage tracing	[68]
HCC; iCCA	Hepatic Progenitor Cell	Wild type C57BL/6; *pGCDNsam-Bmi1-IRES-EGFP*; *pGCDNsam-β-catenin-IRES-EGFP*; *CSH1-shBmi1-EF-1α-EGFP*	Isolation and in vitro transduction of H-CFU-Cs; subcutaneous and intra-splenic cell transplantations	[73]
		*Alb-Cre*; *Sav*^*flx/flx*^; *Mst1*^*flx/flx*^; *Mst2*^*flx/flx*^	GEMM	[78]
Hepatoma		*Alb-Cre*; *WW45*^*flx/flx*^;	GEMM	[79]
HCC		*Alb-Cre*; *Mst1*^*–/–*^; *Mst2*^*flx/flx*^	GEMM	[80]
HCC; iCCA		*Sox2-Cre*; *CAGG-Cre*^*ER*^; *Mst1*^*Δ/Δ*^; *Mst2*^*flx/Δ*^	GEMM	[81]
		*Alb-Cre*; *Mx1-Cre*; *Ad-Cre*; *Nf2*^*flx/flx*^; *B6;129Gt[ROSA]26Sor*^*tm1Sho*^*/J*;	GEMM; lineage tracing; partial hepatectomy; cell transplantation	[82]
iCCA or HCC		*Alb-Cre*; *R26*^*lox-stop-lox-NICD-ires-nls-EGFP*^ *Retro-c-Myc/Akt*	GEMM; cell transplantation	[85]
		*Alb-Cre*; *LSL-Kras*^*G12D*^; *Hnf4α*^*flx/flx*^; *LSL-IDH2*^R172K^;	GEMM; DEN	[87]
	Hepatic Progenitor Cell; hepatoblast; or hepatocyte	NOD/SCID; *Hras Luciferase/EGFP*; *SV40LT-mCherry*; *sh-c-Myc*	Cell transplantation; lentiviral transduction	[91]
HCC; iCCA	Hepatocyte	*Alb-Cre*; *p19Arf*^*−/−*^; *Rosa26*^*loxP-mTom-stop-loxP–mGFP*^; *Mlkl*^*flx/flx*^; TLR2^*−/−*^; TLR3^*−/−*^; TLR4^*−/−*^; TLR7^*−/−*^; TLR9^*−/−*^; *CMV-SB*; *pCaMIA- c-Myc-IRES-myrAKT1*; *pCaMIA-c-Myc-IRES-Nras*^G12V^; *pCaggs-Tbx3*; *pCaggs-Prdm5*	HTVI; GEMM;	[63]
HCC	Hepatocyte/ Hepatic Progenitor Cell	*Alb-Cre*; *Atg5*^*flx/flx*^*; Atg7*^*flx/flx*^*;* *Pten *^*flx/*+^; *Rosa26*^*loxP-mTom-stop-loxP–mGFP*^;	GEMM	[92]

*CCl*_*4*_ carbon tetrachloride, *CMV* cytomegalovirus, *DEN* Diethylnitrosamine, *GEMM* genetically-modified mouse models, *H-CFU-Cs* hepatic colony-forming units in culture, *HFD* high fat diet, *HTVI* hydrodynamic tail-vein injection, *MUP-uPA* major urinary protein- urokinase-type plasminogen activator, *STZ* streptozotocin, *TAA* thioacetamide, *TCPOBOP* 3,30,5,50-tetrachloro-1,4-bis(pyridyloxy)benzene, *SB* Sleeping Beauty

## Hepatocytes as CoO

Hepatocytes comprise approximately 60% of liver cells and 80% of the total liver mass [16] making this cell type the prime suspect of serving as a CoO, especially for HCC. They are organized into functional units called lobules, in which hepatocytes can be divided into functionally specialized zones; zone 1 located closest to the portal trials comprising portal veins, hepatic arteries, and bile ducts; zone 3 surrounding the central vein, and zone 2 located between zone 1 and zone 3 [16]. Hepatocytes are highly specialized and metabolically active epithelial cells, which on the one hand are responsible for the uptake of nutrients and xenobiotic detoxification, and on the other hand manage the production of a large number of proteins, lipids, and bile acids [17]. As a consequence of high metabolic activity and exposure to gut-derived bacterial products coming through the portal vein, hepatocytes generate large amounts of Reactive Oxygen Species (ROS), which can damage DNA and eventually cause hepatocyte death. In addition, hepatocytes are susceptible to infection by hepatitis viruses, HBV, and HCV. Chronic inflammation caused by these infections leads over time to cycles of hepatocyte death and compensatory proliferation, which eventually results in the development of chronic liver disease, fibrosis, and cirrhosis; well-recognized risk factors for the development of HCC [18]. Finally, the work generated in various laboratories in recent years indicated that under physiological circumstances, hepatocytes are able to self-renew and regenerate liver without engaging cells with stem or progenitor properties [19, 20]. Taken together, all these data point to a possibility that the hepatocyte may be a supposed CoO of HCC. This hypothesis has been rigorously tested by several investigators using mouse models.

Early studies utilizing chemical carcinogenesis in rodent models shed some light on the issue of CoO in HCC. Even fully mature hepatocytes located near a central vein in a pericentral zone 3 can undergo oncogenic transformation in mice and rats exposed to Diethylnitrosamine (DEN). DEN is an *N*-nitroso compound that after oral or parenteral administration is converted into dimethylnitrosamine, an alkylating metabolite responsible for the formation of DNA adducts and mutagenesis. DEN is mainly metabolized in zone 3 hepatocytes due to their expression of an enzyme cytochrome 2E1 (Cyp 2E1). This in effect may cause hepatocyte death [21], but surviving hepatocytes may accumulate mutations in some potent oncogenes (e.g., *Braf* or *Hras*) leading to their transformation [22, 23]. Unlike other carcinogens, DEN does not lead to an early elevation in α-fetoprotein (AFP), a marker of immature hepatocytes (hepatoblasts) and purported hepatic progenitor cells (HPC) [24–27]; instead, transformed AFP-positive hepatocytes appear in DEN-treated livers only later and are followed by the development of AFP-positive HCC [28]. Therefore, DEN appears to promote hepatocarcinogenesis by transforming only fully mature Cyp 2E1-expressing hepatocytes, thus inevitably limiting the process of tumor initiation to this cell type. However, due to this limitation, these studies did not definitively exclude the possibility that some rare stem/progenitor cells or even cholangiocytes could serve as CoO for HCC. In fact, in a rat model of DEN-induced hepatocarcinogenesis, the appearance of OV-6 positive HPC (also called oval cells in rodents) was easily detectable [29]. Moreover, it is possible that, due to a high plasticity of cancer cells (discussed in detail below), they may lose their initial identity (hepatocyte) and acquire traits characteristic of precursors (stem/progenitor cells) or even different lineages (cholangiocytes).

Therefore, although the chemically induced hepatocarcinogenesis was a robust and reproducible process, its toxicity and possible induction of oncogenic transformation of other than zone 3 residing hepatocytes precluded the definitive determination of a cell type from which HCC originated, and highlighted the need for more precise and less toxic tools. It was the advent of a genetic lineage tracing strategy in mice that enabled a more accurate establishment of the cellular hierarchy in the liver and addressing the issue of CoO in liver cancer.

### Principles of lineage tracing

Lineage tracing enables the identification of progeny of a single cell, which is marked in such a way that the mark is transmitted to all of the cell’s progeny, resulting in a set of labeled clones. Lineage tracing provides information on the location of the progeny of marked cellsand their differentiation status. For a good lineage tracer, the key features are that it should not change the properties of the marked cell, its progeny, and its neighbors. Critically, the label must be passed on to all the offspring of the founder cell, should be retained over time, and should never be transferred to neighboring cells [30]. In a lineage tracing based on genetic recombination, a recombinase enzyme is expressed in a cell or tissue-specific manner to activate the expression of a reporter gene, thus permanently genetically labeling all progeny of the marked cells. The most frequently used lineage tracing system is based on a cell type-specific expression of Cre recombinase combined with a reporter gene, whose transcription is prevented by a STOP cassette flanked by loxP sites, which are precisely recognized and excised by Cre recombinase [30]. Excision of the STOP cassette in Cre-expressing cells initiates transcription of the reporter gene and permanent labeling of this cell and its progeny. Temporal and spatial control of recombination can also be inducible. In this approach, Cre is fused to the human estrogen receptor, leading to the generation of a Cre^ER^ fusion protein. In the absence of an activating ligand (estrogen or an antiestrogen tamoxifen), Cre^ER^ remains inactive in the cytoplasm, while in the presence of the ligand, Cre^ER^ translocates to a cell nucleus and catalyzes the excision of the STOP cassette and initiates transcription of the reporter gene [31]. Later, in order to reduce Cre^ER^ activation by endogenous estrogens and to lower the concentration of tamoxifen used, new mutated generations of Cre^ER^ (Cre^ERTam^, Cre^ERT^, and Cre^ERT2^) have been produced [32–34]. These technical advances set the stage for the experimental determination of cells that initiate liver cancer in mouse models of the disease with a much greater precision than a chemical approach and the expression of cytological markers.

## Lineage tracing-based studies supporting hepatocytes as CoO in liver cancer

Due to the close histological similarity between hepatocytes and cells that make up the bulk of HCC tumors, hepatocytes were prime suspects responsible for the appearance of tumorigenesis, and the majority of studies published so far point to this cell type as a CoO in liver cancer, especially HCC (Table 1). Accordingly, one study which implicated hepatocytes as CoO in HCC used lineage tracing based on tamoxifen-inducible *Sox9-Cre*^*ERT*^ in R26R^*YFP*^–expressing reporter mice [35]. In the liver, the *Sox9* promoter is active primarily in progenitor cells located near portal triads, and to a lesser extent in the pericentral region in so-called hybrid hepatocytes, which possess stem cell properties and regenerative potential. To gauge the relative contribution of liver progenitor cells, *Sox9*^*ERT*^; R26R^*YFP*^–based tracing was performed in mice challenged with a chemical carcinogen DEN, genetically modified *MUP-uPA* mice fed high fat diet (HFD) [36], or STAM model of diabetes-promoted HCC [37]. In this approach, progenitor cells and hybrid pericentral hepatocytes were marked with YFP protein. However, strikingly, no YFP positive cells were detected in hyperproliferative lesions and well-differentiated tumor nodules in either model. These data strongly argued against progenitor cells or hybrid hepatocytes and instead pointed to fully differentiated hepatocytes as CoO in HCC.

Another approach to determine the CoO in HCC utilized a tamoxifen-inducible Cre driven by an *Osteopontin* (*Opn*) promoter and R26R^*YFP*^ reporter [38]. The combined mutant mice (*Opn-Cre*^*ERT2*^) expressed the *Cre*^*ERT2*^ allele specifically in the progenitors and biliary cells. Treatment of adult mice with tamoxifen lead to translocation of *Cre*^*ERT2*^ to the nucleus and activation of *Yfp* expression and permanent labeling of *Opn*-expressing cells with YFP. Importantly, YFP faithfully marked cells in the LPC/biliary compartment, not hepatocytes, Stellate cells, or Kupffer cells. Induction of HCC with the administration of DEN led to the development of typical HCC tumors with enlargement of the hepatocytic plates, high proliferative index, disruption of the reticulin network, absence of portal tracts, focal expression of α-fetoprotein (AFP) and OPN. However, none of the tumors examined expressed detectable levels of the YFP protein, which was only present in cholangiocytes and cells that formed the ductular reaction surrounding the tumors. Virtually the same results were obtained showing lack of YFP positive HCC when *Opn-Cre*^*ERT2*^ allele was replaced with another biliary-specific *Cre* allele, *Keratin19*-*Cre*^*ERT2*^ (*K19-Cre*^*ERT2*^). In complementary experiments, hepatocytes were specifically labeled and traced with an intravenous injection of adeno-associated virus serotype 8 (AAV8) that expressed Cre recombinase under the control of hepatocyte-specific thyroxin-binding globulin (TBG) promoter (AAV8-Tbg-Cre) in combination with Rosa26^loxP-mTom-stop-loxP–mGFP^ (mTom-mGFP) or Rosa26^loxP-stop-loxP–ZsGreen1^ (ZsGreen) Cre reporter mice. Again, DEN administration led to the development of typical HCC tumors, but this time close to 100% of these tumors showed GFP expression, that signified they were derived from mature hepatocytes. Furthermore, the tumors also expressed characteristic HCC markers (*Gpc3*, *Golm1*, *mKi67*, *Tff3,* and *Tspan8*). When DEN was combined with treatment with a hepatotoxin CCl_4_, feeding with a choline-deficient, 0.15% ethionine-supplemented (CDE) diet or a 0.1% 3,5-diethoxycarbonyl-1,4-dihydro-collidin–supplemented (DDC-supplemented) diet, the expression of the progenitor and hepatoblast markers *alfa-fetoprotein*, *H19* and a stem cell marker *Prominin1* was much higher than in the group treated with DEN only, yet this time virtually all HCC were GFP positive. Taken together, these results unequivocally pointed to a differentiated hepatocyte, but not a progenitor cell or cholangiocyte, as the CoO in DEN-induced HCC. Thus, these lineage tracing based studies were congruent with DEN studies, and supported the hepatocyte as a CoO in mechanistically different murine models of HCC.

However, these results could have been skewed by the fact that DEN was still utilized in these studies, and as stated above, inadvertently initiated hepatocarcinogenesis in zone 3 hepatocytes. To correct for this possibility, non-genotoxic HCC mouse models were used [38]. In the first model, liver carcinogenesis was triggered by the genetic deficiency of a *phospholipid export pump Mdr2*, (*Mdr2*^KO^ mice). The absence of MDR2 led to spontaneous inflammation followed by fibrosis and finally to the development of HCC, thus reproducing the sequence of events that lead to human HCC [39]. The second model was based on hepatocyte-restricted *Pten* deletion achieved by tail vein injection of AAV8-Tbg-Cre. *PTEN* (*Pten* in mice) is a tumor suppressor gene that shows reduced expression in approximately 50% of human HCC cases, and its loss is inversely correlated with patients survival [40]. In both models, the tumors that developed were in their majority typical, GFP positive HCC. In contrast, deletion of *Pten* in the biliary compartment using *K19-Cre*^*ERT2*^ did not produce any tumors with HCC characteristics, demonstrating that, at least in this model, cells in the LPC/biliary compartment did not serve as a CoO for HCC. Importantly, all cells expressing progenitor cell markers (AFP, A6, and K19) that were found in the HCC of AAV8-Tbg-Cre-injected mice were GFP positive, and therefore most likely derived from malignant hepatocytes that had undergone a process of dedifferentiation and acquisition of progenitor cell marker expression, but not from *bona fide* progenitor or biliary compartments.

The finding of hepatocyte as a CoO in HCC was confirmed in another mouse model, *hURI-tetOFF*^hep^ [41]. In this model, the human unconventional prefoldin RPB5 interactor (hURI) is expressed specifically in hepatocytes, and NAD^+^-deficiency-induced DNA damage causes multistep liver tumorigenesis mimicking human disease, with the development of focal nodular hyperplasia, regenerative nodules, nonalcoholic steatohepatitis (NASH), HCA, and HCC [42, 43]. To establish a link between hepatocytes and CoO in liver cancer, *hURI-tetOFF*^hep^ mice were crossed with serum albumin (SA)*Cre*^*ERT2*^-expressing and R26R^*YFP*^ reporter lines, generating progeny in which all hepatocytes and their descendants were marked with permanent expression of YFP after tamoxifen injection. All livers and developing tumors were YFP positive, indicating that hepatocytes contributed to hepatocarcinogenesis. However, in some cases, while the peritumoral tissue was YFP positive, the tumors were highly heterogeneous mixtures of YFP positive and YFP negative hepatocytes, and some tumors were even completely YFP negative. A similar trend was observed in (SA)*Cre*^*ERT2*^/R26R^*YFP*^ mice treated with DEN. The conclusion of this study was that hepatocytes can participate in the initiation of liver tumorigenesis, but are not the exclusive CoO of liver tumors in models involving cessation of hepatocyte proliferation and their death. Furthermore, in R26^*mTmG*^ reporter mice infected with AAV8-Tbg-Cre and treated with DEN+CCl_4_, or in *Mdr2*^KO^ mice crossed with the ZsGreen reporter line and infected with AAV8-Tbg-Cre, HCC tumors were exclusively derived from hepatocytes. Further indirect evidence supporting the contribution of hepatocytes as CoO in HCC came from studies tracking the fate of HNF1β-positive biliary cells in mice with tamoxifen-inducible expression of dTomato driven by *Hnf1bCre*^*ER*^. In this model, the expression of *Hnf1bCre*^*ER*^ was restricted to the biliary compartment, as determined by co-staining for HNF1β, CK19, or SOX9 one week after a single injection of tamoxifen [44]. Treatment with DEN or crossing with *Mdr2*^KO^ mice resulted in liver tumorigenesis, with tumors displaying the classical features of HCC; expression of AFP, Golgi protein 73, a proliferation marker Ki-67, and loss of collagen IV expression. However, these tumors never expressed dTomato, clearly indicating that they were derived from hepatocytes rather than biliary cells.

These conclusions were consistent with the results obtained in a study investigating DEN+CCl_4_ induced liver tumorigenesis in R26^*YFP*^ mice injected with AAV8-Tbg-Cre to label all hepatocytes, or in mice with the *Forkheadbox protein L1-Cre/R26*^*YFP*^ (*Foxl1-Cre/R26*^*YFP*^) in which *Cre* is expressed in a subset of HPCs [45]. In the context of AAV8-Tbg-Cre infection followed by DEN+CCl_4_, all tumors that appeared to be HCC were uniformly positive for YFP, indicating that they were derived from hepatocytes, although they were also positive for progenitor cell markers, Sox9, Cd133, Cd44 and delta-like 1 homolog. In a complementary approach, the appearance of HPC and their contribution to the development of HCC were investigated. Treatment of *Foxl1-Cre/R26*^*YFP*^ mice with DEN+CCl_4_ led to the accumulation of FOXL1/YFP double positive HPC, even prior to the induction of liver tumors. However again, HCC tumors were YFP negative confirming that they were derived from hepatocytes, not *Foxl1*-expressing HPC. Similar results were obtained in another mouse model of HCC based on DEN+TCPOBOP treatment. TCPOBOP is an agonist for the constitutive androstane receptor [46, 47]. It promotes hepatocarcinogenesis through multiple mechanisms, including activation of the c-Myc-FoxM1 pathway and up-regulation of antiapoptotic proteins [48–50]. Also in this case, lineage tracing of hepatocytes using AAV8-Tbg-Cre in R26^*YFP*^ mice showed that all HCC tumors were positive for YFP, thus hepatocyte-derived. On the contrary, the same treatment scheme in *Foxl1-Cre*/*R26*^*YFP*^ mice led to the development of only YFP negative HCC tumors.

Furthermore, Tschaharganeh et al. [51] provided compelling evidence that transduction of differentiated hepatocytes with *Yap*, a potent liver oncogene with increased activity in both HCC and iCCA [52], together with knockdown of *trp53* tumor suppressor, lead to dedifferentiation of hepatocytes and acquisition of stem and progenitor cell characteristics. A plasmid encoding tetracycline-regulated *Yap* gene, together with GFP reporter gene was administered to trace the transduced cells, along with a plasmid encoding shRNA targeting and potently inhibiting a *trp53* tumor suppressor, or control vectors were delivered using a hydrodynamic tail vein (HDTV) injection. This method allows for selective transduction of mature hepatocytes, as validated with morphological criteria and lineage tracing methodologies [53]. The livers of Yap/shp53-transduced mice showed foci of small, undifferentiated, GFP positive tumor cells that exhibited markers of bipotential liver progenitor cells. Tumors that formed appeared aggressive with many mitotic figures and an invasive growth pattern. The emergence of progenitor cell phenotypes was associated with the re-expression of gene coding for *Nestin*, a progenitor cell marker [54]. Mechanistically, the p53 protein blocked *Nestin* promoter activity by repressing the Sp1/3 transcription factors. Importantly, Nestin appeared to be functionally involved in liver tumorigenesis, since its knockdown completely blocked tumor formation. Together, these data indicated that *Yap* overexpression and *trp53* downregulation conspired to induce hepatocyte dedifferentiation and tumorigenesis through de-repression of the *Nestin* gene. Interestingly, combining p53 knockdown with activation of other clinically important oncogenic pathways, Wnt or Notch altered in a significant percentage of human HCC or iCCA, respectively [55], led to the formation of tumors with HCC or iCCA characteristics, respectively [51]. Therefore, p53-deficient hepatocytes can produce tumors that adopt characteristics of distinct cell fates depending on the initiating and cooperating oncogenic events.

As many patients suffering from viral hepatitis develop iCCA frequently containing p62-positive hyaline inclusions [56] that are otherwise specific for injured or malignant hepatocytes, it was suggested that iCCA can also be derived from hepatocytes [57–59]. This issue has been addressed by two studies that used lineage tracing in mice with genetically marked hepatocytes or cholangiocytes [53, 60]. The first study [53] used a hydrodynamic tail vein injection (targeting only hepatocytes) of plasmids encoding the intracellular domain of the NOTCH1 receptor (NICD; Myc-tagged), AKT (HA-tagged) along with sleeping beauty transposase to help their genomic integration. Malignant tumors that developed as a result displayed either a ductular or cystic phenotype reminiscent of human iCCA. The presence of Myc-tag and HA-tag testified that they were uniformly derived from NICD/AKT-expressing cells. The survey of lineage-specific markers revealed that emerging tumors specifically expressed biliary markers (CK-19 and EpCAM), but not hepatocyte markers (Major urinary protein and AFP). The fact that many of these iCCA tumors developed in the central region of the lobule, where hepatocytes predominate, led to the hypothesis that hepatocytes, not cholangiocytes, were their CoO. To validate this hypothesis, genetic lineage tracing was used, in which *R26*^*YFP*^ reporter mice were transduced with AAV8-Tbg-Cre and subsequently injected with NICD/AKT/sleeping beauty plasmids to initiate tumorigenesis. Positive EYFP immunostaining clearly indicated that the CoO of the iCCAs was a hepatocyte, not a cholangiocyte. Furthermore, the iCCAs expressed the biliary markers, Sox9, CK-8, and mucin 1 (Muc1), but were negative for the hepatocyte marker Mup. This study raised the possibility that iCCA under some circumstances may originate from hepatocytes, not cholangiocytes.

The second study confirming the role of hepatocyte as a CoO in iCCA [60] used mice expressing *Cre*^*ERT2*^ from an *albumin* promoter, or expressing *Cre*^*ERT2*^ from a *CK19* promoter, which were crossed to *R26*^*LacZ*^ or *R26*^*YFP*^ reporter mice. This approach enabled the tracking and visualization of hepatocytes or cholangiocytes and their progeny after tamoxifen administration. To chemically induce iCCA, the *Alb-Cre*^*ERT2*^;*R26R*^*lacZ*^ and *CK19-Cre*^*ERT2*^;*R26R*^*lacZ*^ mice were treated with thioacetamide (TAA). After 30 weeks of TAA treatment, all mice developed primitive ductules and neoplastic nodules with typical characteristics of iCCA (cytoplasmic mucin granules and CK19 positivity), however these nodules lacked HNF4α expression, eliminating the possibility that they were HCC with pseudoglandular differentiation. Staining for X-gal or a biliary cell marker EpCAM demonstrated that *Alb-Cre*^*ERT2*^;*R26R*^*lacZ*^ mice, but not *CK19-Cre*^*ERT2*^;*R26R*^*lacZ*^ mice contained ductules with double positive cells, suggesting that they were derived from hepatocytes rather than cholangiocytes. Mechanically, TAA treatment led to the appearance of CK19 positive cells near the central veins. These cells expressed YFP in *Alb-Cre*^*ERT2*^; *R26R*^*YFP*^ mice, but not in *CK19-Cre*^*ERT2*^;*R26R*^*YFP*^ mice, indicating that hepatocytes residing next to central veins were initially converted into CK19 positive biliary lineage cells. Furthermore, genetic gain-of-function and loss-of-function experiments using *Alb-Cre*^*ERT2*^; *R26R*^*Notch/*+^ (in which hepatocytes overexpressed an intracellular, constitutively active fragment of the Notch1), and *Alb-Cre*^*ERT2*^;*Hes*^*flx/flx*^ mice (in which hepatocytes lacked the Notch effector Hes1), respectively, demonstrated that increased Notch signaling resulting from TAA administration was able to transdifferentiate hepatocytes into cells that formed iCCA, thus giving a mechanistic insight into how hepatocytes could generate iCCA. Together, these two studies suggested that increased NOTCH signaling can reprogram hepatocytes into iCCA generating cells.

The strict requirement of Notch signaling for hepatocyte reprogramming and iCCA formation was confirmed in other studies utilizing *R26R*^*YFP*^–based, AAV8-Tbg-Cre infection-initiated hepatocyte lineage tracing combined with tumorigenesis triggered by HDTV injection of activated forms of AKT (myr-AKT) and Yap (Yap^S127A^) oncogenes [61]. Interestingly, in these settings, blocking of the canonical Notch signaling with an inhibitory protein dnRBPJ or a conditional deletion of the Notch2 receptor, but not Notch1 receptor, resulted in the absence of iCCA development and exclusive formation of hepatocellular adenoma and HCC-like lesions. Moreover, iCCA developed when Notch signaling was activated in mice together with overexpression of the intracellular domain of Notch receptor (NICD) and expression of constitutively active myr-AKT [62]. These tumors displayed the expression of the biliary fate determinants *Sox9* and *Yap1* and interestingly, conditional deletion of each of these genes separately significantly reduced the burden of iCCA, while combined deletion of both genes completely abolished tumor formation. Furthermore, iCCA generation in this model was dependent on the YAP1-mediated engagement of its downstream transcriptional coactivator TEAD, which subsequently increased the expression of an epigenetic modulator, DNA methyltransferase 1 (DNMT1), and eventually endowed hepatocytes with biliary epithelial cell characteristics. This work provided further insight into our understanding of hepatocyte transdifferentiation into biliary cells through NOTCH pathway activation.

The commitment of an oncogenically transformed hepatocyte towards either HCC or iCCA may also be affected by the microenvironment. Seehawer et al. [63] addressed this issue by HDTV injection of transforming oncogenes into *p19Arf*^*−/−*^ mice. The injected vectors encoded oncogenic mouse *Myc* and *Nras*^G12V^, or mouse *Myc* and human *AKT1*, together with a plasmid encoding the sleeping beauty transposase [63]. All the resulting tumors turned out to be HCC, as revealed by typical solid or trabecular growth pattern of this tumor type and a strong expression of HNF4α. However, strikingly, when the same plasmids were transfected focally using in vivo electroporation (Epo) method [64], most tumors arising were either iCCA or combined iCCA–HCC. Lineage tracing studies in *Alb-Cre*; *R26R*^*mT/mG*^; *p19Arf*^*−/−*^ mice confirmed that both HCC and iCCA were derived from hepatocytes. These results suggested that the mode of plasmid delivery can profoundly influence the directionality of tumor differentiation, HCC vs. iCCA. A deeper dissection of the mechanisms revealed that three days after the procedure both methods increased hepatocyte death, but through different mechanisms. Whereas HDTV primarily induced apoptosis, as evaluated by the number of cleaved Caspase-3-positive cells, Epo produced little apoptotic cells, but greatly increased the levels of RIPK3 and phosphorylated MLKL, established markers of necroptosis [65]. Furthermore, specific inhibition of necroptosis induction with necrostatin-1 or hepatocyte-specific *Mlkl* knockout, switched the phenotype of tumors arising after Epo from iCCA towards HNF4α-positive HCC. It is well recognized that cells undergoing necroptosis release damage-associated molecular patterns that can shape the microenvironment via the activation of pattern recognition receptors (e.g. Toll-like Receptors, TLRs) and subsequent cytokine release from immune cells [66]. Accordingly, mice that lacked primarily TLR2 and TLR4 expression phenocopied the switch from iCCA to HCC observed after necroptosis inhibition. Furthermore, the necroptotic tumor environment appeared to epigenetically (through inducing different chromatin availability states) regulate the expression of two transcription factors TBX3 and PRDM5, which then cooperatively determined lineage commitment in primary liver cancer. Thus, this study strongly implicated the role of tumor microenvironment in the determination of hepatocyte faith; wherein cytokines released from immune cells in response to TLR activation epigenetically rewire the expression of faith-determining transcription factors and eventually dictate the commitment of transformed hepatocytes towards iCCA instead of HCC. In conclusion, all these studies point to hepatocyte as a cell from which liver cancer originates, even though they used different initiators of tumorigenesis (chemical vs genetic); modes of Cre gene expression (endogenous promoters vs viral transfer; expressed constitutively vs inducibly), and used different markers of cell differentiation. However, at the same time these studies demonstrate that the nature of transforming oncogenic events, and even the tumor microenvironment can influence the CoO in liver cancer. The findings of this study are also of great importance for efforts aiming at understanding liver cancer CoO, since they clearly indicate that even relatively small technical differences in the way through which transforming agents are introduced into the liver may immensely impact the final results.

## Cholangiocytes as CoO

The introduction of pro-tumorigenic alterations in the cholangiocyte was reported to generate only iCCA, but not HCC, raising the possibility of a lack of the transdifferentiation ability of this cell type. The ability of cholangiocyte to serve as a CoO for iCCA was documented using several mouse models utilizing lineage tracing. Interestingly however, even infliction of similar injury and cell lineage tracing models may lead to different conclusions. For example, to investigate the nature of CoO in iCCA, Guest et al. exposed *CK19-Cre*^*ERT2*^;*R26R*^*YFP*^;*trp53*^*flx/flx*^ mice to TAA in order to induce iCCA formation [67]. The difference in genetic modifications between this model and the one used by Sekiya S et al. discussed above [60] was the additional deletion of the *trp53* gene. However, 26 weeks of TAA treatment of *CK19-Cre*^*ERT2*^; *R26R*^*YFP*^*; trp53*^*flx/flx*^ mice produced multifocal tumors that were uniformly YFP positive confirming their biliary origin. Importantly, no cells were dually positive for YFP and the mature hepatocyte marker Cyp2D6, indicating the lack of expression of *CK19-Cre*^*ERT2*^ in hepatocyte lineage. On the other hand, tumor cells showed expression of the Notch 1 receptor, which frequently co-localized with YFP, and was co-expressed with a mature cholangiocyte marker, M3 acetylcholine receptor. Given this evidence, and the fact that the *CK19-Cre*^*ERT2*^; *R26R*^*YFP*^*; trp53*^*flx/flx*^ mice did not show hepatocyte lineage labeling, it was concluded that the YFP-positive tumor cells originated from cholangiocytes rather than hepatocytes.

Another level of complexity to the issue of liver cancer CoO is added by the impact of the level and timing of gene expression. An intriguing insight into the effect of gene dosage on CoO in liver cancer came from a study investigating the impact of *Kras*^*G12D*^ activation in the mouse liver in the context of a different number of gene copies encoding *Pten* tumor suppressor [68]. In this study, liver specificity of genetic modifications was ensured by crossing mice containing *Kras*^*LSL*^ and a different copy number of conditional *Pten* alleles (*Pten*^*flx*^) with *Alb-Cre* mice. All the resulting mice developed liver tumors, but their histological type was dependent on the number of intact *Pten* alleles retained. When no *Pten* allele was deleted (*Kras*^*G12D*^*;Pten*^+*/*+^), hepatocellular dysplasia was formed, with no abnormalities in the biliary system; in contrast, deletion of both *Pten* alleles (*Kras*^*G12D*^*;Pten*^*∆/∆*^) produced only iCCA; while deletion of just one *Pten* allele (*Kras*^*G12D*^*;Pten*^*∆/*+^) produced both HCC and iCCA. To elucidate whether mature hepatocytes may be the origin of iCCA induced by *Kras* activation and *Pten* deletion, *Alb-Cre*^*ERT2*^ mice were used. *Alb-Cre*^*ERT2*^*; Kras*^LSL^*; Pten*^*flx/flx*^ mice were injected with tamoxifen 8 weeks after birth to induce gene recombinations. All tumors that appeared in these mice 3 months later were HCC, but not iCCA. Interestingly, when gene recombination was induced on postnatal day 10 (P10), all tumors that formed were iCCA; the discrepancy that could be explained by exclusive activation of *Alb-Cre*^*ERT2*^ in hepatocytes at 8 weeks, but in both hepatocytes and cholangiocytes on P10. When the *CK19-Cre*^*ERT2*^ allele was used to induce genetic recombination in the biliary compartment, mice developed only premalignant papillary ductal lesions in the periportal areas. In all models these tumor types were confirmed to express lineage-specific markers. Thus, this study indicated that at least in the settings of activated *Kras* oncogene in murine livers, the amount of *Pten* alleles and the timing of transgene modification can, to a large extent, dictate whether a liver cancer CoO will give rise to HCC or iCCA.

Taken together, these studies indicated that deletion of *trp53* tumor suppressor in the biliary lineage combined with chemical damage inflicted specifically to the biliary system resulted in iCCA with a cholangiocyte serving as a CoO. In addition, they pointed to the importance of gene dosage and time of genetic modifications on the lineage commitment of a transformed cell.

## Hepatic progenitors as CoO

The fact that, depending on the type of initiating event and model used, both hepatocytes and cholangiocytes could initiate the formation of HCC and/or iCCA, and given that combined HCC-iCCA, the type of liver cancer that presents both HCC and iCCA histological subtypes, frequently displays stem cell characteristics [69] raised a question about the ability of Hepatic Progenitor Cells (HPC) [70–72] to undergo malignant transformation and to generate liver tumors. This question is of clinical importance given the particularly poor prognosis of this tumor type. To address this possibility, Chiba et al. isolated stem/progenitor cells from murine fetal livers based on cell surface expression of CD45 and Ter-119 markers [73]. Subsequently, these cells were transduced in vitro with retroviruses expressing stem cell renewal-associated genes, *Bmi1*, or mutant *β-catenin*. Immunocompromised mice transplanted with the engineered HPCs developed tumors consisting of albumin positive hepatocytes and glandular structures composed of CK7 positive cholangiocytes. Interestingly, the tumors contained nests of bipotential cells expressing both albumin and CK-7. Furthermore, the HCC and iCCA that formed were intimately intermingled and exhibited no clear borders. Together, these experiments illustrated that the oncogenic transformation of embryonic liver stem/progenitor cells could lead to the outgrowth of mixed tumors closely resembling human combined HCC/iCCA and argued for the ability of HPC to act as liver cancer CoO, although the relevance of fetal liver progenitor cells as CoO of adult-onset mixed HCC-iCCA remains unclear.

Additional lines of evidence implicating HPCs as CoO in liver cancer came from studies on mouse models with a deranged Hippo pathway. This pathway is evolutionary conserved and participates in regulation of organ size, stem and progenitor cell renewal and expansion, regeneration, and tumorigenesis [74–76]. The Hippo pathway plays a tumor suppressor role through phosphorylation of transcriptional coactivators YAP and TAZ thus inhibiting their nuclear translocation and stimulation of downstream tumor-promoting genes [77]. Inactivation of the Hippo pathway in murine liver through deletion of the serine-threonine protein kinases *mst1* and *mst2* or their adapter protein *sav1/WW45* increased liver size and hepatocyte proliferation, and resulted in the development of HCC and iCCA [78], or formation of hepatomas positive for oval cell marker A6 [79]. The fact that both major types of liver cancer occurred in these mice and that an abundant oval cell reaction was detected in the periportal regions of these mice, pointed to an oval cell (HPC) as the CoO in these models. However, another study utilizing *Alb-Cre*- or Adenovirus-mediated Cre delivery reported that deletion of *mst1* and *mst2* only caused HCC formation, with no iCCA development or oval cell proliferation [80]. Interestingly, when the *mst1* and *mst2* genes were deleted using *Cre* expressed from a tamoxifen-inducible, ubiquitously-expressed promoter (*CAGGCre-ER*), HCC and iCCA developed in the absence of oval cell accumulation [81]. These studies showed the importance of the Hippo pathway in liver tumorigenesis, yet at the same time they emphasized the impact of context, i.e., timing of genetic modification and the nature of promoter sequences which drive Cre recombinase expression, as major determinants of the generated liver cancer types and their CoO.

Deletion of *Nf2/Merlin*, another member of the Hippo/Yap pathway, was also reported in mouse liver to induce both HCC and iCCA uniformly containing transitional cellular morphologies, with hepatocytic and biliary features [82]. In mice with liver-restricted deletion of *Nf2/Merlin*, overt tumorigenesis was preceded by robust proliferation of oval cells that were positive for pan-Cytokeratin, A6 and Cd34 antigens. Importantly, however, the impact of *Nf2* deletion was independent of the Hippo pathway, as intracellular localization of the YAP protein remained unchanged. Instead, Epidermal Growth Factor (EGF) receptor signaling persisted in oval cells and hepatoblasts even at high cell density, indicating that these cells failed to undergo a contact-dependent inhibition of proliferation. On the contrary, deletion of *Nf2* in the livers of *Nf2*^*flx/flx*^ mice through adenovirus-mediated *Cre* delivery, or crossing with interferon-inducible *Mx1-Cre* mice [83], did not produce tumors of any type. This result could be attributed to the quiescent nature of the mature liver, as the stimulation of a robust proliferative response following partial hepatectomy (PHx) reinstalled an abundant oval cell reaction and the subsequent development of HCC and iCCA. Furthermore, transplantation of *Nf2*^*–/–*^ hepatoblasts into the hepatectomized livers of wild type mice also produced HCC and iCCA. Thus, the regenerative stimulus provided by PHx appears to specifically unleash the overproliferation of otherwise quiescent *Nf2*^*–/–*^ liver progenitors.

Along the same lines, liver-restricted activation of the NOTCH pathway through liver-specific expression of an intracellular domain of Notch1 (*Rosa26Notch1IC*) [84] led to the formation of small cell clusters and gland-like structures with an epithelial appearance [85]. These cells stained positive for biliary/hepatocyte as well as stem cell markers; a typical finding for cells that show characteristics of combined hepatocyte and cholangiocyte differentiation, and often arise from transformed HPCs [86]. When transplanted subcutaneously into immunosuppressed mice, primary *NICD*-overexpressing liver fragments formed iCCA positive for CK7 and CK17, and a typical desmoid reaction of the surrounding tissue. These results suggested that expression of *NICD* in HPC could induce their differentiation toward the biliary lineage and promoted their malignant transformation over time. Interestingly, when progenitor cells were stably transduced with vectors encoding c-Myc and a constitutively active form of AKT, tumors that formed displayed an undifferentiated histology of HCC/hepatoblastoma. These results indicated that the expression of the intracellular domain of NOTCH lead to the development of iCCA, and that transforming oncogenes could dictate the directionality of HPC differentiation and the type of arising liver tumors.

HPC has also been identified as a CoO in iCCA arising as a result of the expression of mutant isocitrate dehydrogenases 1 and 2 (IDH1/2; mutIDH1/2) in the liver [87]. While wild type IDH1/2 are enzymes that catalyze oxidative decarboxylation of isocitrate to α-ketoglutarate and CO_2_, their mutant forms exhibit a neomorphic catalytic activity responsible for the production of an oncometabolite, 2-hydroxyglutarate (2-HG) [88, 89]. The expression of mutant *IDH1/2* was found in a significant percentage of human iCCA [90]. To test the impact of mutIDH1/2 on the differentiation of HPC and liver tumorigenesis, Saha et al. [87] generated mice with tetracyclin-inducible expression of mutant *IDH2* (*IDH*^R172K^). They showed that *IDH*^R172K^ abrogated the differentiation potential of HPCs towards hepatocytes. This effect was mediated by blocking the expression of a gene encoding *HNF4-α*, a hepatocyte lineage specification transcription factor. In effect, hepatocyte differentiation potential of HPC was disabled, while biliary differentiation potential was retained; a phenomenon that has been shown to be mediated by the generation of 2-HG by *IDH*^R172K^. In fact, DEN treatment of mice with liver-specific *HNF4-α* deletion led to progenitor cell expansion and subsequent development of iCCA, but not HCC. Furthermore, *IDH*^R172K^ and *Kras*^G12D^ double-mutant murine livers showed a greatly accelerated tumorigenic potential compared to *Kras*^G12D^-only mutant livers, with metastatic tumors expressing iCCA histopathology and expression of a biliary marker, CK19, but lacking expression of an HCC marker, HepPar1. All *IDH*^R172K^/*Kras*^G12D^ mutant livers contained areas of proliferating oval cells positive for SOX9 and CK19. Thus, *IDH*^R172K^ and *Kras*^G12D^ can cooperatively drive the generation of iCCA preceded by HPC expansion.

The question of CoO of liver cancer has been systematically addressed by an elegant work by Holczbauer et al. [91]. To directly establish the ability of different liver cells to become cancer-initiating CoO (i.e. to acquire cancer stem cell, CSC, properties), primary HPC, lineage-committed hepatoblasts (HB), and differentiated adult hepatocytes (AH) were transduced with transgenes encoding oncogenic *Hras* and *SV40 Large T antigen LT* (*SV40LT*). As it turned out, all three cell types could acquire CSC properties and became liver cancer CoO, as defined by an increase and/or acquisition of the side cell population fraction, CD133 cell surface expression, and the ability to grow as self-renewing spheres. Importantly, HPC-generated CSC formed tumors after subcutaneous implantation with the highest efficiency, while AHs were the most resistant to oncogenic transformation and tumor formation. This is consistent with the notion that HPCs need significantly fewer steps than their more differentiated progeny to acquire CSC properties and generate tumors. Furthermore, regardless of which liver cell type was transformed, tumors were highly metastatic, moderately-to-poorly differentiated, and with varying contribution of HCC-, iCCA- and epithelial-to-mesenchymal transition (EMT)-like phenotypes. However, the relative contribution of HCC-, iCCA- and EMT-like tumors differed depending on which cell type was oncogenically transformed; HCC dominated in AH-derived tumors, iCCA in HB-derived tumors, and EMT-like type in HPC-derived tumors. Furthermore, strong and uniform expression of the progenitor/biliary markers CK19 and A6 were detected regardless of tumor CoO, and a significant proportion of differentially expressed genes was associated with EMT, consistent with the high metastatic propensity of these tumors. Furthermore, AH-derived tumors showed the highest number of differentially expressed genes compared to parental hepatocytes. These results conclusively showed that any cell type belonging to the hepatic epithelial lineage can be a target of oncogenic transformation and can acquire a common CoO properties via activation of various cell type-specific pathways. Moreover, this study suggests that transformation of HPC may circumvent the necessity to first dedifferentiate more mature cell types (hepatocytes and cholangiocytes); however, it leaves unanswered the question of how relevant the transformation of fetal HPC is to the adult hepatocarcinogenesis.

Deregulation of intrinsic tumor suppressor mechanisms has also been shown to contribute to HCC formation through the generation of HPC. As an example, Barthet et al. [92] reported that inhibition of autophagy through *Alb-Cre*-driven conditional deletion of central autophagy genes 5 (ATG5) or ATG7, combined with heterozygous deletion of *Pten,* accelerated HCC formation. Mechanistically, hepatocytes from *Alb-Cre;Atg5*^*flx/flx*^*;Pten*^*flx/*+^ and *Alb-Cre;Atg7*^*flx/flx*^*;Pten*^*flx/*+^ mice displayed a high propensity to dedifferentiate to HPC and form a ductular reaction determined by accumulation of the HPC markers SOX9, CK19, and panCK. The use of *R26*^*mTmG*^ reporter mice and AAV8-Tbg-Cre infection-based lineage tracing strategy showed that SOX9-positive ductular cells expressed GFP protein signifying that they were derived from hepatocytes, and suggesting that loss of autophagy enabled dedifferentiation of hepatocytes towards HPC and subsequent HCC formation. Furthermore, the expression of Hippo pathway effectors, YAP and TAZ, turned out to be critical for this process, since their deletion significantly decreased the ductular reaction and completely abrogated tumor formation. Thus, autophagy is emerging as a tumor suppressive mechanism that prevents hepatocyte dedifferentiation and tumorigenesis.

## Mechanisms responsible for cell faith change during liver carcinogenesis

### Dedifferentiation

The most obvious process that can contribute to misinterpretation of the data on CoO in liver cancer is dedifferentiation. This process implies the reversal of fully differentiated cell characteristics to less differentiated ones, typical of cells at earlier stages in the same lineage [93]. In physiological situations, dedifferentiation is frequently associated with tissue regeneration and allows for replenishment of cells that have been lost. However, dedifferentiation can be hijacked by cancer cells to produce cells with stem/progenitor cell properties. In HCC, several pathways can be involved in the dedifferentiation of hepatocytes into cells with HPC characteristics. One possible player in this process is the YAP protein, an effector of the Hippo pathway. YAP activation has been shown to contribute to the formation of a ductular reaction through hepatocyte dedifferentiation during liver regeneration in mice [94] and, clinically, YAP protein levels in patients were positively correlated with the dedifferentiation of HCC tumors [95]. Furthermore, autophagy deficiency in hepatocytes promoted their dedifferentiation into biliary-like progenitor cells that led to HCC through activation of YAP and TAZ [92].

Another contributor to dedifferentiation in liver cancer cells are loss-of-function mutations of a tumor suppressor protein p53, an alteration commonly found in HCC [96] and iCCA [97], or its inactivation due to interactions with protein products of the HBV [98] and HCV [99]. The activity of the p53 protein is well documented to prevent cell dedifferentiation. In embryonic stem cells, the p53 protein binds to promoter sequences and suppresses Nanog expression, a gene required for ESC self-renewal and dedifferentiation [100]. The p53 protein is also involved in the expression of differentiation-associated genes [101]. Similarly, wild type p53 restrains induced pluripotent stem cells dedifferentiation and reprograming in vitro [102]. Congruently, the livers of *trp53*^*−/−*^ mice showed an accumulation of blast-like cells containing scant cytoplasm and small nuclei typical of ductular progenitor cells, suggesting that their differentiation process was deregulated [103]. Further studies confirmed this observation and added more mechanistic explanations. Thus, inactivation of *trp53* expression and activation of the Yap oncogene in murine livers produced tumors with characteristics of undifferentiated progenitor cells through repression of *Nestin* (a progenitor cell marker) promoter activity [51], suggesting that Nestin is necessary for the dedifferentiation of hepatocytes and their reprograming to malignant progenitors. These data are consistent with other studies implicating p53 in the regulation of cell plasticity. For example, loss of p53 contributes to the development of glioblastoma in mice [104]. Furthermore, on the flip side, it was shown that p53 re-expression in embryonic carcinoma cells deficient in p53 or HL-60 promyelocytic leukemia cells was shown to trigger their differentiation [105, 106] and may partly explain the association between the presence of p53 mutations and stem cell characteristics in certain cancers [107, 108]. Disruption of other tumor suppressive pathways may also enable dedifferentiation and thus complicate the interpretation of lineage tracing studies. In particular, Rb-Arf axis was implicated in regulating differentiation in skeletal muscle [109] and thyroid cancer [110], and c-Myc oncogene upregulation as part of ESC signature enabled the dedifferentiation of adult hepatocytes into cancer initiating cells in a mouse model of liver cancer [91]. In conclusion, alterations in oncogenes or tumor suppressor genes commonly found in cancer, may induce dedifferentiation of malignant cells, and thus complicate the task of identifying liver cancer CoO.

### Transdifferentiation

As mentioned above, on the basis of histological examination, it is reasonable to suppose that HCC derives from hepatocytes and iCCA from cholangiocytes. However, this assumption may be false, since, as mentioned above, HCC and iCCA share some common risk factors, particularly infections with HBV, HCV, and cirrhosis. Furthermore, the combined HCC-iCCA often contains the same genetic alterations in both components. This observation raises the possibility that fully differentiated liver cells may lose their cell identity and acquire morphological and transcriptional characteristics of a different liver cell lineage. In the context of chronic tissue injury, this process is often referred to as transdifferentiation and resembles metaplasia, which is precipitated by constant exposure to noxious and frequently inflammatory stimuli and is considered a premalignant state [111, 112]. In the liver, such a situation is not without a precedence; it is known that hepatocytes can change their identity and become cholangiocytes through transdifferentiation during the course of chronic liver injury. Cell transdifferentiation classically involves dedifferentiation followed by activation of an intrinsic genetic program that allows redifferentiation into a new cell lineage [93]. In this respect, it was shown that damage to biliary tract elicited by biliary toxins and bile duct ligation in rats [113] lead to a large-scale conversion of hepatocytes into biliary ductules. Similarly, genetic lineage tracing in mice with liver-specific expression of a NICD (*Rosa*^*NICD*^), led to the generation of new cholangiocytes through transdifferentiation of hepatocytes [114]. In this study, YFP positive cells (hepatocytes) acquired biliary morphology and expression of cholangiocyte markers (OPN, A6, SOX9 and CK19), in a process that involved an intermediate state characterized by the expression of hepatocyte and cholangiocyte markers. Similar findings were reported by Tarlow et al., who additionally showed that the ability to undergo hepatocyte-cholangiocyte transdifferentiation was conserved in humans [115]. However, in these studies a significant percentage of hepatocyte-derived biliary cells returned to hepatocyte morphology after the initial injury had subsided [115, 116], and hepatocyte-derived ductules did not contribute to the drainage of bile [117]. Therefore, despite the highly similar morphology and marker expression, hepatocyte-derived and cholangiocyte-derived cells retained memory of their respective parental lineages and were functionally different. However, it should be emphasized that in these experimental systems the biliary network was fully developed, making it likely that mature cholangiocytes were regenerating the injured bile ducts and thus relieving pressure on hepatocytes to transdifferentiate into cholangiocytes. The circumstances may be different in situations where the generation of normal, functional intrahepatic biliary network from biliary progenitors is impossible due to genetic deficiencies. One such example is Alagille syndrome, an autosomal dominant, complex multisystem disorder, which in the liver is characterized by paucity of the bile ducts, cholestasis, and eventual liver failure. In the vast majority of cases the Alagille syndrome is caused by impaired NOTCH signaling [118]. To mimic human disease, Schaub et al. generated a mouse model of Alagille syndrome based on a liver-specific deletion of NOTCH signaling effectors, *RBPJ*, and the deletion of a transcription factor *HNF6* [119]. The *Alb-Cre*; *Rbpj *^*f/f*^; *Hnf6 *^*f/f*^ mice were severely cholestatic because they lacked peripheral bile ducts at birth. However, surprisingly, on day 120 of postnatal life, more than 90% of mice developed a functional biliary tree. To test the hypothesis that these postnatally formed bile ducts were derived from hepatocytes, *R26NZ*^+*/*+^ mice were used, in which EGFP expression is initiated by flippase-mediated recombination. Indeed, after injection of AAV-Ttr-Flp viruses the newly formed peripheral bile ducts were shown to be EGFP positive indicating their hepatocyte origin. In contrast to metaplastic biliary cells [115–117], they showed acetylated tubulin-marked primary cilia, expressed mature biliary lineage markers (CK19 and EPCAM), and a marker of biliary function, somatostatin receptor 2, thus demonstrating that peripheral cholangiocytes derived from hepatocytes were authentic and fully mature. Dissection of molecular mechanisms revealed that pSMAD3 levels and the expression of genes related to TGF-β signaling were upregulated in hepatocyte-derived cholangiocytes, and liver-restricted deletion of TGF-β receptor 2 caused truncation or total absence of the biliary network in *Alb-Cre*; *Rbpj*^*f/f*^; *Hnf6*^*f/f*^ mice, while overexpression of a constitutively-active TGF-β receptor 1 had an opposite effect. Similar mechanisms have also been shown to work in patients with Alagille syndrome [119]. Together, this study indicated that under extreme conditions, hepatocytes can go beyond the reversible metaplasia and transdifferentiate into legitimate, fully functional cholangiocytes in a process governed by TGF-β signaling. Although it is currently unknown whether a similar process may occur during liver carcinogenesis, this could be a plausible mechanism of the generation of iCCA from hepatocytes in the context of chronic liver damage. Interestingly, by analogy, hepatocytes may be derived from cholangiocytes when their regenerative abilities are blunted. Accordingly, it was shown that in the context of liver-specific ablation of β1-integrin or liver-specific overexpression of *Cdkn1a* encoding p21 cell cycle inhibitor (modifications that impaired hepatocyte proliferation and regenerative potential), liver regeneration continued due to creation of hepatocytes through transdifferentiation of cholangiocytes [120]. However, whether a similar process could contribute to HCC formation has not yet been shown in human samples or experimental animals, but if present, it would definitively complicate determination of the liver cancer CoO.

### Epithelial-to-mesenchymal transition (EMT)

EMT is a process in which epithelial cells lose their epithelial characteristics (e.g. loss of E-Cadherin, an epithelial marker expression) and acquire mesenchymal traits (e.g. gain of N-Cadherin, a mesenchymal marker expression) which is accompanied by changes in cell morphology and behavior (e.g. gaining of fibroblastic morphology and more migratory properties) [121]. This process is reversible, meaning that cells may reverse to a more epithelial state in a process of a Mesenchymal-to-Epithelial Transition (MET). EMT may be broadly divided into three types: type I—which occurs in embryogenesis during gastrulation and gives rise to mesenchymal cells which will later undergo MET to generate secondary epithelia; type II—occurring later during organogenesis and generating fibroblasts colonizing interstitial spaces; it may also result from prolonged injury or inflammation leading to organ fibrosis; and type III—which generates metastatic cancer cells able to relocate to distant places and form secondary outgrowths. TGF-β family of secretory cytokines is one of the most prominent and well-established inducers of EMT [122]. Moreover, NOTCH signaling has been shown to induce EMT in cancer cells and increase their aggressiveness [123], and after liver transplantation it may contribute to cholangiocyte EMT [124]. Although both TGF-β and NOTCH were, as stated above, implicated in the generation of cholangiocytes and iCCA through transdifferentiation of hepatocytes, the interrelationship between EMT and dedifferentiation/transdifferentiation processes is not clear. However, EMT has been shown to contribute to dedifferentiation of cancer cells and, under certain conditions, to the generation of cells with cancer stem cell properties [125]. Consistently, highly metastatic and metaplastic breast cancer encompasses both glandular and nonglandular components, the latter of which derives from the mesenchymal differentiation of the epithelium and displays EMT markers expression [126]. Since, as stated above, the process of transdifferentiation is usually preceded by dedifferentiation, it seems reasonable that EMT may also play a role in transdifferentiation. In fact, sequential EMT-MET cycles have been shown to participate in the generation of several tissues and organs, e.g. the heart [127] and the kidney [128]. However, the evidence for the involvement of EMT in liver organogenesis is scarcer; it was reported that cultured hepatocytes from neonatal rat livers can undergo an EMT, as revealed by the loss of specific differentiation markers, the gain of a migratory phenotype, and the replacement of typical hepatocyte cytokeratins by vimentin. The features of EMT were also found in hepatocytes and progenitor cells isolated from rodent and human fetal livers [129]. Interestingly, TGF-β signaling was shown to participate in organogenesis, and specifically in the liver, high activin/TGF-β signaling near the portal veins is required for proper differentiation of biliary cells. The Onecut family transcription factors, HNF-6 and OC-2, inhibit activin/TGF-β signaling in the parenchyma, allowing normal hepatocyte lineage commitment. In the absence of Onecut factors, the shape of the activin/TGF gradient is altered, and the hepatoblasts differentiate into hybrid cells that display characteristics of both hepatocytes and biliary cells. Thus, a gradient of activin/TGF signaling modulated by Onecut factors is required to segregate the hepatocyte and biliary lineages [130].

Further evidence for the involvement of EMT in transdifferentiation comes from studies on the generation of induced pluripotent stem cells (iPSC) from fully differentiated cells. Sequential EMT-MET has been implicated in the reprogramming of mouse embryonic fibroblasts (MEF) into iPSC. The transition of MEF from a mesenchymal state to an epithelial state has been recognized as a required step during the early phases of reprogramming, which was greatly impaired when EMT was enforced or MET was inhibited. Mechanistically, pretreatment or short treatment with TGF-β decreased proliferation rate and significantly increased reprogramming efficiency, but longer treatments decreased the reprogramming efficiency. Although sequential EMT-MET has not been reported during differentiation and transdifferentiation, it is reasonable to suggest that inducing sequential EMT-MET may, under certain conditions facilitate cell fate conversions [131].

Additionally, in the mature organism, EMT is frequently induced in tissues that undergo constant damage. It was shown that hepatocytes derived from human cirrhotic livers chronically infected with HBV displayed features of EMT and stained positive for phosphor-SMAD2 and SNAIL indicative of ongoing TGF-β signaling [132]. In in vitro experimental systems, the treatment of primary mouse hepatocytes or a hepatocyte cell line AML12 with TGF-β1 resulted in changes consistent with the induction of EMT: decreased levels of E-Cadherin, increased levels of Vimentin, increased expression of transcription factor *Snail1*, and enhanced deposition of collagen type I; all these changes were largely reversed by knockdown of SMAD pathway using *Smad4*-targeted siRNA [133]. These findings were confirmed by another study that used hepatocytes derived from CCl_4_-injured cirrhotic murine livers [134]. Zeisberg et al. [135] were the first to demonstrate the evidence for hepatocyte EMT in vivo. Using *LacZ* reporter activated by albumin-driven Cre, they showed that CCl_4_ challenge led to the accumulation of fibrotic cells, 45% of which were double positive for a fibrotic cell marker FSP-1 and a hepatocyte marker β-galactosidase indicating their hepatocyte origin. Treatment of primary hepatocytes isolated from *Alb-CRE/R26*^*LacZ*^ mice with TGF-β1 upregulated FSP-1 expression yielding FSP-1/ β-galactosidase double-positive hepatocytes. Thus, these studies raised the possibility, that at least some of the liver fibrotic cells might be generated through transdifferentiation of hepatocytes. However, this conclusion has been refuted by Taura et al. [136], who showed that in genetically engineered mouse models, where hepatocytes were marked by *Alb-Cre*-initiated *LacZ* expression and *collagen type I*-expressing cells by *collagen α1(I)* promoter-driven GFP expression, cells isolated from CCl_4_-treated livers never showed double-positivity for GFP and β–galactosidase. Furthermore, in liver sections from CCl_4_-treated mice GFP-positive areas were coincident with the fibrotic septa and never overlapped with β–galactosidase-positive areas. These studies established the ground for a highly controversial field of EMT as a possible source of fibroblasts (discussed in [137–139]). However, the fact that during embryonic development TGF-β governs the biliary differentiation of hepatoblasts raises the possibility that the same mechanisms can be used during transdifferentiation of hepatocytes to initiate the iCCA in chronically damaged livers of patients. In this regard, EMT has been shown to confer resistance to therapy in both HCC [140] and iCCA [141].

### Limitations of lineage tracing

With all the advantages that lineage tracing approach provides, one should keep in mind that it possesses some limitations that should be considered for prudent interpretation of the results. For example, a single recombinase may only target one population of cells, and in constitutively expressed *Cre* models, many of the lines can express *Cre* in cells of interest at one point and in some other cells at a later time point, complicating interpretation of the end results. Moreover, sometimes the promoter of interest can be weakly activated in other cell types, which is considered an ectopic expression. This ectopic expression can sometimes lead to genetic labeling, which is unwanted and unintentional tracing, and may lead to misinterpretation of some cell fate studies [142]. In addition, Cre recombinase may also cause gene mutations, thus inducing developmental abnormalities or even embryonic lethality, and tamoxifen may cause off-target effects, e.g., damaging the gastric mucosa [143]. In order to circumvent these limitations, novel more complex modes of lineage tracing were developed [144, 145].

Another major drawback of lineage tracing strategy is its limited utility to trace CoO of liver cancer in human patients. Although we have learned a lot about the nature of CoO using lineage tracing from rodent studies, the utility of this knowledge to a clinical situation in which cancer develops over the years in the background of chronic liver disease caused by viral infection or fatty liver disease is uncertain. In humans, however, experimental lineage tracing would be unethical and technically challenging. Nevertheless, recent studies have demonstrated that it is now possible to trace cell lineages using natural variations in nuclear and mitochondrial DNA, as well as variations in DNA methylation status [146]. These emerging technologies could revolutionize the field of lineage tracing and the determination of liver, and other cancers’ CoO.

## Conclusions

In general, experimental studies utilizing both chemical and genetic means to induce liver carcinogenesis, and employing genetic lineage tracing as a way to track the faith of transformed cells, found that basically any type of liver epithelial cells may become transformed and initiate tumorigenesis. These seemingly contradictory results may be simply explained by the specificity of Cre expression; the cells which initiate cancer are the ones in which Cre is expressed and activates pro-tumorigenic genetic modifications. Moreover, while it seems that cholangiocytes can only generate iCCA, hepatocytes and HPCs can give rise to both HCC and iCCA depending on the transforming conditions. More specifically, activation of the NOTCH pathway in hepatocytes is particularly effective in skewing hepatocytes towards generation of iCCA. In addition, gene dosage (e.g. *Pten*) and even microenvironment can profoundly influence the choice whether HCC or iCCA will be generated. In this regard, necrotic cell death and the ensuing inflammation seem to encourage the generation of iCCA from transformed hepatocytes. Since all of these factors can influence cell faith change, they should be taken into consideration when trying to properly establish the CoO in liver cancer. Processes that impact cell faith determination, such as dedifferentiation, transdifferentiation, and epithelial-to-mesenchymal transition, play an important role in cancer formation and emerge as new cancer hallmarks [147]. In this regard, it is possible that hepatocytes, cholangiocytes, and their stem/progenitor cells may directly, without changing their cellular identity, undergo oncogenic transformation and initiate tumorigenesis. Moreover, oncogenic transformation of one cell type (e.g. hepatocyte by *Notch* oncogene) may induce its transdifferentiation into another type (e.g. cholangiocyte) and thus lead to iCCA formation, in which case a hepatocyte is serving as a CoO for iCCA. Importantly, some oncogenes are biased towards a specific cell type, whereas others may change cell identity through, e.g. transdifferentiation, and initiate tumors histologically typical of another cell lineage. Finally, transformation of hepatocytes and cholangiocytes may require prior dedifferentiation to progenitor cells (which in this case serve as bona fide cancer stem cells), and only these cells may subsequently generate HCC or iCCA depending on which cell faith they acquire. It is important to emphasize that dedifferentiation, transdifferentiation, and EMT are not mutually exclusive, and may participate in cell faith change simultaneously or sequentially. The exact mechanisms through which liver epithelial cells change their developmental faith necessitate experimental validation in the future, but are important from a clinical standpoint; determining how exactly liver tumorigenesis proceeds may elucidate whether it is enough to eliminate lineage committed cancer cells, or whether it is necessary to target cancer stem cells as well.

Another unresolved issue is the nature of liver cancer CoO in human patients. Whereas the issue of CoO in rodent models has been extensively addressed, it should be kept in mind that, although highly sophisticated, these models are artificial systems substantially different from the situation found in human liver cancer patients. Therefore, the question of human liver cancer CoO is still open and will have to be addressed in the future. Currently, largely due to methodological constraints, much less is known about the cell types from which human liver cancer arises, particularly in the context of chronic viral hepatitis and nonalcoholic fatty liver disease. These conditions are more difficult to model in rodents, but identification of the liver cancer CoO in more clinically relevant situations could inform better therapies and preventive measures. The development of such models will be a significant challenge for the future.

## Data Availability

Not applicable.

## Abbreviations

2-HG: 2-Hydroxyglutarate
AAV8: Adeno-associated virus serotype 8
AFP: α-Fetoprotein
AH: Adult hepatocytes
BECs: Biliary epithelial cells
CDE: Choline-deficient, ethionine-supplemented diet
CoO: Cell-of-origin
CSC: Cancer stem cell
Cyp 2E1: Enzyme cytochrome 2E1
DDC-supplemented: 3,5-Diethoxycarbonyl-1,4-dihydro-collidin–supplemented diet
DEN: Diethylnitrosamine
DNMT1: DNA methyltransferase1
EMT: Epithelial-to-mesenchymal transition
Epo: Electroporation
ESC: Embryonic stem cells
GFP: Green fluorescent protein
HB: Lineage-committed hepatoblasts
HBV: Hepatitis B virus
HCC: Hepatocellular carcinoma
HCV: Hepatitis C virus
HDTV: Hydrodynamic tail vein
HFD: High fat diet
HPC: Hepatic progenitor cells
hURI: Human unconventional prefoldin RPB5 interactor
iCCA: Intrahepatic cholangiocarcinoma
IDH1/2: Isocitrate dehydrogenases 1 and 2
iPSC: Induced pluripotent stem cells
MEF: Mouse embryonic fibroblasts
MET: Mesenchymal-to-Epithelial Transition
Muc1: Mucin 1
NAFLD: Non-alcoholic fatty liver disease
NASH: Non-alcoholic steatohepatitis
NICD: Intracellular domain of Notch receptor
OPN: Osteopontin
P10: Postnatal day 10
PHx: Partial hepatectomy
PLC: Primary liver cancer
ROS: Reactive Oxygen Species
shRNA: Short hairpin RNA
TAA: Thioacetamide
TAZ: Transcriptional coactivator with PDZ-binding motif
TBG: Thyroxin-binding globulin
TCPOBOP: 1,4-Bis-[2-(3,5,-dichloropyridyloxy)] benzene
YAP: Yes-associated protein
YFP: Yellow fluorescent protein
