# Correction: Current view of liver cancer cell-of-origin and proposed mechanisms precluding its proper determination

**DOI:** 10.1186/s12935-023-02875-0

**Published:** 2023-02-21

**Authors:** Tomasz Gromowski, Veronika Lukacs-Kornek, Jaroslaw Cisowski

**Affiliations:** 1 Department of General Biochemistry, Faculty of Biochemistry, Biophysics and Biotechnology, Jagiellonian University, Krakow, Poland; 2 Institute of Experimental Immunology, University Hospital of the Rheinische Friedrich-Wilhelms-University, Bonn, Germany

**Correction: Cancer Cell International (2023) 23:3** **https://doi.org/10.1186/s12935-022-02843-0**

In this article [1], the statement in the Funding information section was incorrectly given as 'This work was supported by a grant from the National Science Foundation, Poland (Opus-19 2020/37/B/NZ4/02533) to JC' and should have read “JC is funded by the National Science Centre (Grant Number 2020/37/B/NZ4/02533). VLK is funded by the Deutsche Forschungsgemeinschaft (DFG, German Research Foundation) under Germany’s Excellence Strategy—EXC 2151 – 390873048 and DFG Project number 411345524.”
